# Median Nerve-Neurophysiological Index Correlates With the Survival of Patients With Amyotrophic Lateral Sclerosis

**DOI:** 10.3389/fneur.2020.570227

**Published:** 2020-10-22

**Authors:** Liu-Qing Xu, Wei Hu, Qi-Fu Guo, Lu-Lu Lai, Guo-Rong Xu, Wan-Jin Chen, Ning Wang, Qi-Jie Zhang

**Affiliations:** ^1^Department of Neurology and Institute of Neurology, First Affiliated Hospital, Fujian Medical University, Fuzhou, China; ^2^Fujian Key Laboratory of Molecular Neurology, Fujian Medical University, Fuzhou, China

**Keywords:** amyotrophic lateral sclerosis, neurophysiological index, median nerve, prognosis, survival

## Abstract

**Objective:** This study aims to explore the association between median nerve-neurophysiological index (NI) and survival of patients with amyotrophic lateral sclerosis (ALS).

**Methods:** A retrospective case series with a prospective follow-up study was performed in 238 patients with ALS. Their clinical profiles and NI were recorded. Kaplan-Meier curves and Cox regression were adopted to perform survival analysis.

**Results:** The median survival time of all ALS cases was 33.0 months. Multivariate analysis showed that older age of onset, shorter diagnostic delay, higher ΔALSFRS-R, and faster progression {NI ≤ 2.15; hazard ratio [HR] = 1.543 [95% confidence interval (CI), 1.136–2.094]} were associated with short survival. NI was correlated with ALSFRS-R at baseline (*r*_s_ = 0.3153; *p* < 0.0001) and ALSFRS-R at different time points of follow-up (*r*_s_ = 0.5127; *p* < 0.0001). The higher NI slope of decline (> 0.25) showed shorter survival compared with the lower group (≤ 0.25; 34.0 vs. 52.0 months; *p* = 0.0003). A predictive model was constructed based on the age of onset, diagnostic delay, median nerve NI, and ΔALSFRS-R. The higher predictive score (> 14) showed significantly shorter survival compared with the lower group (≤ 14; HR = 3.907, 95% CI, 2.857–5.342).

**Conclusion:** Median nerve NI and its slope of decline were predictive of survival of ALS.

## Introduction

Amyotrophic lateral sclerosis (ALS), also known as Lou Gehrig's disease or motor neuron disease (MND), is a chronic, incurable, and fatal motor neuron degenerative disease that affects both upper and lower motor neurons, giving rise to progressive muscle weakness and atrophy. The worldwide incidence of ALS is estimated at 2–5 per 100,000 people, and the peak prevalence is around age 50–75 (1). Death from ALS typically occurs within 3–5 years after the onset, largely due to respiratory failure.

To date, the electrophysiological examination, especially electromyography (EMG) is the predominant accessory test for ALS. Ulnar nerve-neurophysiological index (NI), which was calculated based on the compound muscle action potential (CMAP) amplitude, distal motor latency (DML), and the frequency of F-waves, was reported to be highly associated with the lower motor neuron loss and the strength of abductor digiti minimi muscle (ADM) (2). Moreover, Cao et al. (3) reported that ulnar nerve NI at baseline could be a reliable electrophysiological marker to predict the survival of sporadic ALS patients in China. Clinically, it was found that the thumb abductor muscle was more prone to be involved at the early stage of ALS, which is also defined as “split hand syndrome” (4–6). Until now, the majority of NI descriptions were based on ulnar nerve studies, and the predictive value of median nerve NI was not clarified. Therefore, we set out to describe the median nerve NI at baseline and the decline rate of NI during follow-up, to observe the correlation between median nerve NI and ALSFRS-R score, and to explore the association between median nerve NI and survival in patients with ALS.

## Materials and Methods

### Patients

All the patients with ALS were enrolled from the Department of Neurology, First Affiliated Hospital of Fujian Medical University from December 2014 to September 2019. The patients were followed up every 3–6 months in person. For those patients at later disease stages, the follow-up was performed by telephone. All the patients that were included in the survival analysis were followed up at least more than 24 months. Patients were diagnosed with definite, probable, or possible ALS according to the revised El Escorial criteria (7). We excluded the ALS cases whose time between symptom onset and EMG test was more than 3 years. Patients with ALS additionally presenting with carpal tunnel syndrome, based on refers to the diagnostic criteria of carpal tunnel syndrome (8–10), were also excluded. When ulnar nerve sensory conduction velocity (SCV) and distal motor latency (DML) were normal, while median nerve SCV slows down, or DML is prolonged beyond the 95% confidence interval comparing with age-matched healthy individuals, it is considered to be abnormal. The normal reference value was adopted from Peking Union Medical College Hospital, China. Clinical profiles including demographic characteristics (gender, nationality, height, and weight), age of onset, site of onset, family history, and motor function (Functional Rating Scale-Revised, ALSFRS-R score, range from 1 to 48) were recorded at baseline. Patients who were treated with riluzole (50 mg, twice per day) for longer than 3 months were defined as “use of riluzole.” The serum levels of creatine kinase (CK) and creatinine were collected closest to date of EMG testing (before or at least 3 days after EMG test). This study was approved by the Ethics Board of the First Affiliated Hospital of Fujian Medical University. Written informed consent was obtained from each participant.

### Electrophysiological Testing

The electrophysiological studies were performed by the same board-certified neurologist using Dantec Key point net System (Dantec Biomedical, Denmark). The skin temperature of the examined limbs (including the normal and atrophic side) was maintained above 34°C during the nerve conduction studies, and the room temperature was 25°C. For median nerve motor conduction study, the recording site was at the abductor pollicis brevis (APB) muscle, and the stimulation site was at the wrist, 7 cm proximal to the recording electrode. The recording site and stimulation site for the F-wave test were the same as those of the median nerve motor conduction study with 20 supramaximal stimuli at 0.5 Hz, and the stimulator should be turned around so that the cathode was at the proximal position. The median nerve NI was calculated as M-wave amplitude/DML × F-wave frequency after 20 stimuli). M-wave amplitude was measured from the first negative peak to the next positive peak. DML was the time from the stimulus to the initial CMAP deflection from baseline. F-wave frequency was a measure of the number of the F-waves obtained per the number of stimulations. The median nerve NI of both the left and right side was calculated for each patient, and the higher NI value was adopted for further statistical analysis.

### Statistical Analysis

The diagnostic delay was defined as the time from symptom onset to an identified diagnosis of ALS. Based on the diagnostic delay, patients were divided into two groups: group 1 (shorter than 12 months) and group 2 (longer than 12 months). Body mass index (BMI) values were divided into three groups (< 18.5, 18.5–23.9, ≥ 24) according to criteria based on the Chinese population. According to ALSFRS-R scores at baseline, patients were divided into two groups as higher or lower than 38, referred to the report from Cao et al. (3). Additionally, the disease progression rate was assessed by ALSFRS-R points lost per month (ΔALSFRS-R), which was expressed as “(48-ALSFRS-Rscore)/disease duration from onset to the visit in months.” For survival analysis, the survival time was calculated from the symptom onset to death or tracheostomy. For those cases that were still alive, survival time was calculated from the symptom onset to the last visit (April 2020). All the statistical analysis was carried out by SPSS v.19.0 software (SPSS, Inc., Chicago, IL, USA). Chi-square test was employed to compare the clinical features of ALS patients based on the median nerve NI. Survival analysis was performed using Kaplan-Meier curves and log-rank test. X-tile software was adopted to obtain the cut-off values of ΔALSFRS-R, median nerve NI, ΔNI, and predictive score. Correlations were calculated with the Spearman coefficient (*r*_s_). The univariate and multivariate Cox regression was adopted to identify the variables associated with survival. *P* < 0.05 (two-tailed) was considered statistically significant.

## Results

In total, 334 ALS patients underwent the initial EMG test within 3 years after disease onset. Among them, 25 cases presented with carpal tunnel syndrome and 10 were lost during follow-up. Sixty-one cases were followed up for <2 years. The remaining 238 ALS cases including 14 familial and 224 sporadic cases were recruited to this study for survival analysis. The ratio of male to female was 1.62:1 (147 males and 91 females). According to the diagnostic category, 156 (65.55%) patients were diagnosed as definite ALS, 64 (26.89%) patients were probable ALS, and 18 (7.56%) patients were possible ALS. According to the site of onset, 45 (18.91%) patients had bulbar onset, 124 (52.10%) patients had upper limb onset, and 69 (28.99%) patients had lower limb onset. The mean age at onset (AAO) was 54.8 ± 11.3 years old. The median diagnostic delay was 10 months.

**Figure 1:**
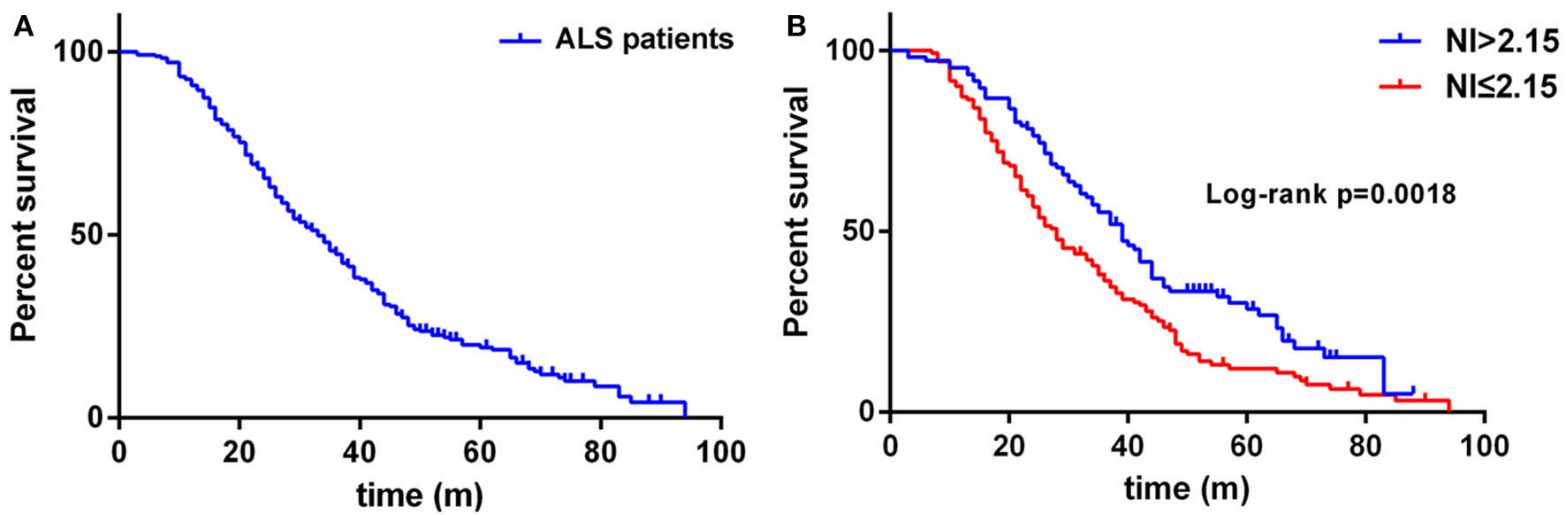
**(A)** Survival analysis for all ALS patients. **(B)** Survival analysis for patients with NI ≤ 2.15 vs. NI > 2.15. NI, neurophysiological index.

Regarding the survival analysis, 192 patients died, 3 received tracheostomy, and 43 (18.07%) were still alive at the last follow-up. The median survival time of all the cases was 33.0 months as shown by Kaplan-Meier analysis (Figure 1A). The median survival for patients with bulbar-onset and spinal-onset ALS was 31.0 and 34.0 months, respectively (*p* = 0.2724). The median value of median nerve NI was 1.80 (0.47–3.45), with a cut-off value of 2.15, as identified using X-tile software. Patients presenting with spinal onset, a lower ALSFRS-R score, or a higher ΔALSFRS-R, showed lower median-nerve NI (Supplementary Table 1). In addition, a significant difference in median survival time was observed between the fast progression group (NI ≤ 2.15) and the slow progression group (NI > 2.15; 28.0 vs. 39.0 months; *p* = 0.0018) (Figure 1B). The results of univariate analyses indicated that gender, AAO, diagnostic delay, BMI, median nerve NI at baseline, ALSFRS-R at baseline, and ΔALSFRS-R were the survival-related variables (Table 1). The multivariate Cox regression analysis was further carried out on the basis of the survival-related variables, as well as site of onset. This indicated that older AAO (> 55; HR = 1.732; 95% CI, 1.275–2.351), shorter diagnostic delay (≤ 12; HR = 1.606; 95% CI, 1.144–2.255), faster progression (NI ≤ 2.15; HR = 1.543; 95% CI, 1.136–2.094), and higher ΔALSFRS-R (> 0.8; HR = 2.495; 95% CI, 1.777–3.501) were correlated with a short survival (Table 2).

**Table 1:** Univariate survival analysis results for ALS patients.

**Variable**	**No (%)**	**Univariate analyses**				
		**β-Value**	**SE**	**Wald**	***p*-Value**	**HR (95% CI)**
**Gender**						
Female	91 (38.24)					1.000
Male	147 (61.76)	0.380	0.152	6.219	0.013	1.463 (1.085, 1.972)
**Age of onset (year)**						
≤ 55	115 (48.32)					1.000
> 55	123 (51.68)	0.715	0.149	23.10	<0.001	2.043 (1.527, 2.735)
**Diagnostic delay (m)**						
> 12	83 (34.87)					1.000
≤ 12	155 (65.13)	0.643	0.153	17.627	<0.001	1.903 (1.409, 2.569)
**Site of onset**						
Lower limb	69 (28.99)					1.000
Upper limb	124 (52.10)	−0.063	0.167	0.142	0.707	0.939 (0.677, 1.303)
Bulbar	45 (18.91)	0.269	0.208	1.675	0.196	1.309 (0.871, 1.969)
**BMI**						
18.5 ~ 24	160 (67.23)					1.000
<18.5	37 (15.55)	0.632	0.192	10.787	0.001	1.881 (1.290, 2.743)
>24	41 (17.22)	−0.562	0.226	6.184	0.013	0.570 (0.366, 0.888)
**Use of riluzole**						
Yes	161 (67.65)					1.000
No	77 (32.35)	0.118	0.152	0.601	0.438	1.125 (0.835, 1.515)
**Median nerve NI**						
>2.15	106 (44.54)					1.000
≤ 2.15	132 (55.46)	0.450	0.147	9.336	0.002	1.568 (1.175, 2.093)
**ALSFRS-R**						
> 38	163 (68.49)					1.000
≤ 38	75 (31.51)	0.610	0.152	16.079	<0.001	1.841 (1.366, 2.482)
****Δ**ALSFRS-R**						
≤ 0.8	132 (55.46)					1.000
> 0.8	106 (44.54)	1.179	0.152	60.048	<0.001	3.251 (2.413, 4.381)

*ALS, amyotrophic lateral sclerosis; ALSFRS-R, amyotrophic lateral sclerosis functional rating scale-revised; BMI, body mass index; NI, neurophysiological index; HR, hazard ratio; CI, confidence interval*.

**Table 2:** Multivariate Cox survival analysis results for patients with ALS.

**Variable**	**β-Value**	**SE**	**Wald**	***p*-Value**	**HR (95% CI)**
**Age of onset (year)**					
≤ 55					1.000
> 55	0.549	0.156	12.374	<0.001	1.732 (1.275, 2.351)
**Diagnostic delay (m)**					
> 12					1.000
≤ 12	0.474	0.173	7.498	0.006	1.606 (1.144, 2.255)
**Median nerve NI**					
> 2.15					1.000
≤ 2.15	0.434	0.156	7.725	0.005	1.543 (1.136, 2.094)
****Δ**ALSFRS-R**					
≤ 0.8					1.000
> 0.8	0.914	0.173	27.94	<0.001	2.495 (1.777, 3.501)

*ALS, amyotrophic lateral sclerosis; ALSFRS-R, amyotrophic lateral sclerosis functional rating scale-revised; NI, neurophysiological index; HR, hazard ratio; CI, confidence interval*.

**Figure 2:**
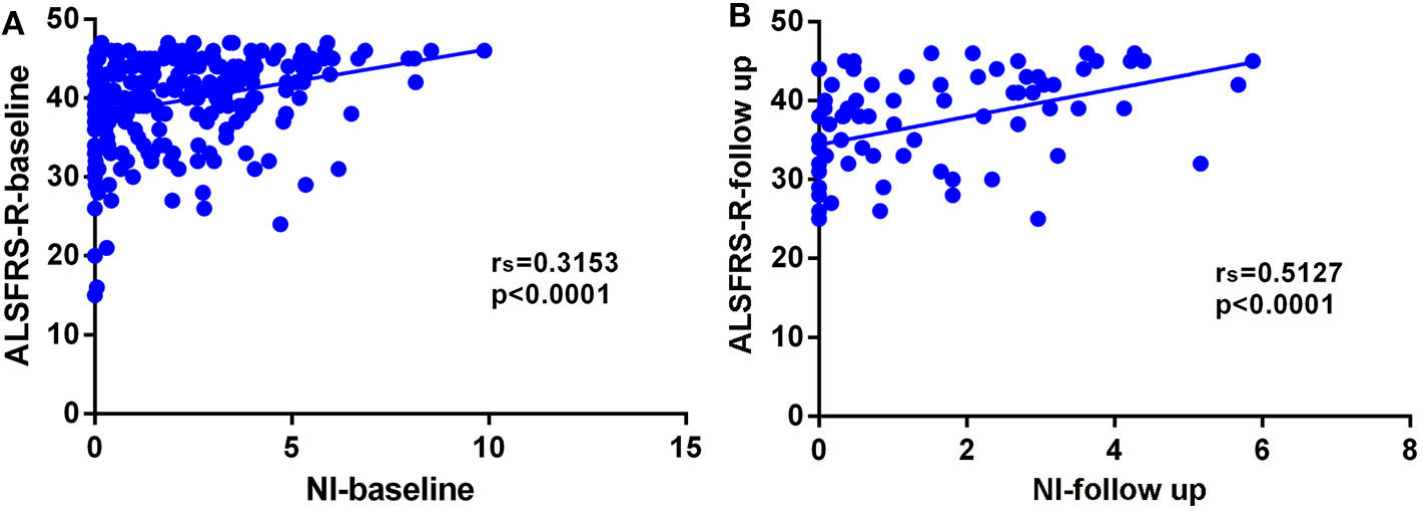
**(A)** Correlation between NI and ALSFRS-R scores at baseline (*r*_s_ = 0.3153, *p* < 0.0001). **(B)** Correlation between NI and ALSFRS-R scores at different time points of follow-up (*r*_s_ = 0.5127, *p* < 0.0001). NI, neurophysiological index; ALSFRS-R, amyotrophic lateral sclerosis functional rating scale-revised; *r*_s_, Spearman coefficient.

**Figure 3:**
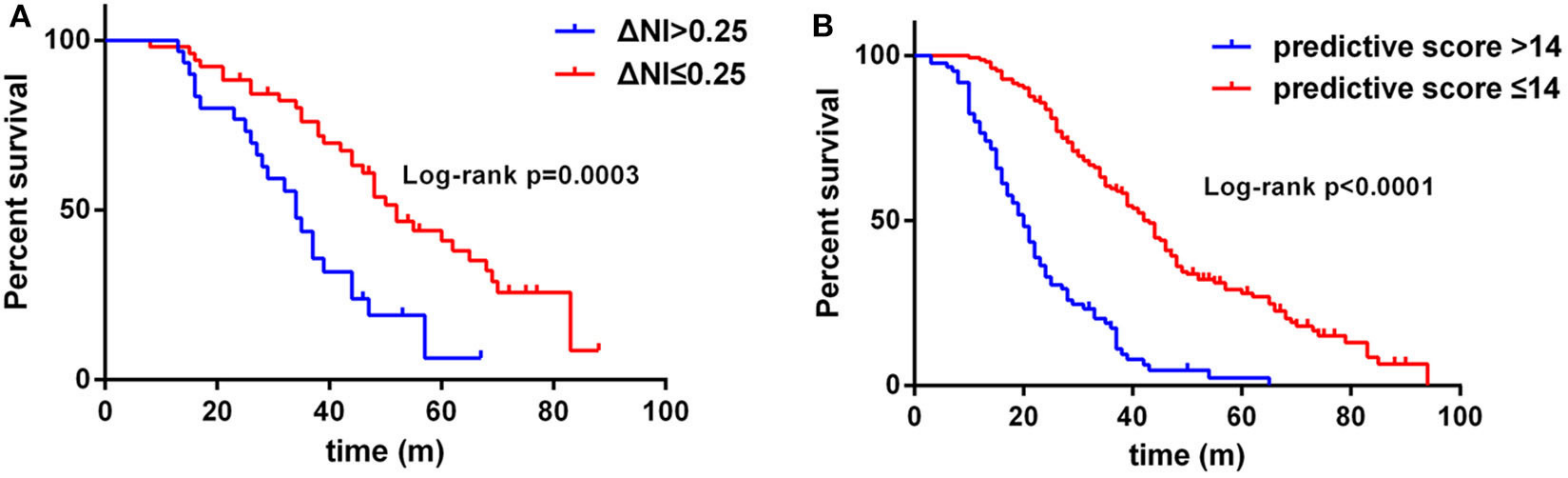
**(A)** Survival analysis for patients with ΔNI ≤ 0.25 vs. ΔNI > 0.25. **(B)** Survival analysis for patients with predictive score ≤ 14 vs. predictive score > 14. NI, neurophysiological index.

In this cohort of patients with ALS, we also explored the association of median nerve NI and CK and creatinine, which are common biomarkers of muscle wasting. It showed that NI was not significantly correlated with serum CK level (*r*_s_ = −0.1761, *p* = 0.0124; Supplementary Figure 1A) and creatinine level (*r*_s_ = 0.09209, *p* = 0.1946; Supplementary Figure 1B). By further exploring the correlation between median nerve NI and disease severity, we found that the median nerve NI was moderately correlated with the ALSFRS-R scores at baseline (*r*_s_ = 0.3153, *p* < 0.0001; Figure 2A). As a retrospective case series study with follow-up, we also calculated the median nerve NI for 81 ALS patients at different time points of follow up, including 39 ones at 3 months, 20 ones at 6 months, 9 ones at 9 months, and 13 ones at more than 9 months. A similar correlation between median nerve NI and ALSFRS-R scores was confirmed (*r*_s_ = 0.5127, *p* < 0.0001; Figure 2B). We further analyzed the change in median nerve NI. The decline in median nerve NI (ΔNI) was calculated by the following formula: (NI at baseline—NI at follow-up)/months between follow-up and baseline. The higher median nerve NI slope of decline (ΔNI > 0.25) showed shorter median survival, compared with the lower group (ΔNI ≤ 0.25) (34.0 vs. 52.0 months, *p* = 0.0003; Figure 3A).

The results of the multivariate Cox regression analysis showed that age of onset, diagnostic delay, ΔALSFRS-R, and median nerve NI were independent predictive factors. A comprehensive predictive model was further constructed based on these variables. The β value of each variable was adopted from the results of multivariate Cox regression (Table 2). The predictive score of each variable equaled to β × 10, and then rounded to an integer (Table 3). The score for older AAO (> 55 years) was 5. The score for a shorter diagnostic delay ( ≤ 12 m) was 5. The score for lower median nerve NI (≤ 2.15) was 4. The score for higher ΔALSFRS-R (> 0.8) was 9. Finally, the total predictive score equaled to the sum score of age of onset, diagnostic delay, ΔALSFRS-R, and median nerve NI. We calculated the predictive score for all ALS patients, and the higher predictive score (> 14) showed significantly shorter median survival compared with the lower group ( ≤ 14; HR = 3.907; 95% CI, 2.857–5.342), and the median survival was 20.0 and 43.0 months, respectively (Chi-square = 84.71; *p* < 0.0001 (Figure 3B).

**Table 3:** Predictive model for ALS.

**Variable**	**β-Value**	**Predictive score**
**Median nerve NI**		
> 2.15		0
≤ 2.15	0.434	4*
**Age of onset (year)**		
≤ 55		0
> 55	0.549	5*
**Diagnostic delay (m)**		
> 12		0
≤ 12	0.474	5*
****Δ**ALSFRS-R**		
≤ 0.8		0
> 0.8	0.914	9*

* *The predictive score of each variable equaled to β × 10, and then rounded to an integer*.

## Discussion

In the present study, we explored the association between median nerve NI and survival in familial and sporadic ALS patients. Firstly, median nerve NI at baseline and survival are correlated in a Chinese patient cohort. Secondly, we identified additional correlations between median nerve NI and ALSFRS-R score, serum CK levels, and serum creatinine levels. Thirdly, we further analyzed the decline rate of median nerve NI in patients with ALS during follow-up. Finally, we also constructed a predictive model based on the variables of the age of onset, diagnostic delay, ΔALSFRS-R, and median nerve NI. Our results indicate that median nerve NI is a predictor of ALS prognosis, even after multiple adjustments of gender, age of onset, site of onset, diagnostic delay, BMI, ALSFRS-R score, and ΔALSFRS-R. Moreover, similar to previous reports, we also confirmed that older AAO, shorter diagnostic delay, and higher ΔALSFRS-R are correlated with a short survival (11–13). These advances in prognostic factor analysis will facilitate the stratification of patient cohorts according to fast and slow disease progression, informing treatment options.

In the clinic, there are several neurophysiological biomarkers of lower motor neuron dysfunction, such as CMAP, motor unit number estimation (MUNE), and motor unit number index (MUNIX) (14). MUNE and MUNIX are methods of assessing the number of motor units innervating a target muscle, which possess the capacity to capture disease progression at the very early disease course (15–17). The fundamental principle of MUNE and MUNIX techniques is to divide the maximal CMAP amplitude by the average surface-recorded motor unit potential, and both of them require the patients to perform a voluntary muscle contraction at various intensity levels (14). Therefore, they are difficult to perform on patients with severe muscle weakness, and results are not reliable when record from muscles innervated by entrapped nerves. The neurophysiological index (NI), calculated from a formula based on CMAP amplitude, distal motor latency, and F-wave persistency, is also a proven sensitive measure for detecting lower motor neuron loss (18, 19). The main advantage of NI lies in requiring little less training and practice, and it is readily available in every neurological department. The NI has been employed in a few clinical trials as an outcome of ALS (20, 21). However, the focal neuropathies due to external compression could largely affect the CMAP amplitude, distal motor latency, and frequency of F-waves. The ulnar nerve NI could be affected by cubital tunnel syndrome, and the median nerve NI could be affected by carpal tunnel syndrome. In the present study, among the 334 patients with ALS, there were 25 cases with coincident carpal tunnel syndrome, with a prevalent frequency of 7.5%. When a patient with ALS presents with carpal tunnel syndrome or cubital tunnel syndrome simultaneously, it is prudent to take all these influencing factors into consideration, including NI, age of onset, diagnostic delay, and disease progression rate.

Currently, the application of NI is mainly based on ulnar nerve. However, at the early stage of ALS, the thumb abductor muscle was involved, which is well-known as “split-hand syndrome,” while the abductor digiti minimi muscle was intact relatively (4). Besides, Imai et al. (22) recently reported that CMAP of the median nerve could be an independent prognostic factor of sporadic ALS cases, and the patients with higher median nerve CMAP amplitude showed a better prognosis. It deserves to describe the predictive value of median nerve NI in disease severity and prognosis. In this study, we found that median nerve NI values were positively correlated with the ALSFRS-R scores at baseline and at different time points of follow-up. A similar correlation between ulnar nerve NI and ALSFRS was previously observed (18). In extension, we also compared the change of median nerve NI for 81 patients at different time points of visit and observed that the NI slope of decline was also correlated with the survival of ALS. The high median nerve NI slope of decline (ΔNI > 0.25) was associated with short median survival. It is well-known that the CMAP amplitude reduces with disease progression. At later stages, the thumb abductor muscle often developed weakness and atrophy severely. The CMAP amplitude was significantly decreased and the F waves were liable to disappear, which could be an obstacle in the calculation and application of median nerve NI. Taken together, we suggest that a combination of ulnar nerve and median nerve NI may favor reflecting the progressive loss of lower motor neurons at different stages of disease course.

The serum CK and creatinine are common markers reflecting the metabolism of muscle. Serum creatinine has previously been reported to be a reliable marker of the severity of disease in patients with ALS (23). A high level of creatinine at baseline was predictive of a slower decline in ALSFRS-R and longer survival (24). The role of CK in the prediction of disease progression in ALS is conflicting. Gibson et al. (25) reported that CK levels were associated with muscle cramping and a low CK correlated with longer survival. Rafiq et al. (26) observed that a higher CK level was associated with longer survival. For majority of ALS cases, the serum levels of CK were normal or mildly elevated with a wide distribution (27). In the present study, we have not observed a significant correlation between median nerve NI and serum level of CK and creatinine. The relationship between NI and CK, creatinine also needs to be confirmed in further studies.

There were some limitations in this study. Firstly, due to a relatively small sample size from a single research center, we have not performed the subgroup survival analysis based on family history (familial vs. sporadic) and the site of onset (bulbar-onset vs. spinal-onset). According to previous studies, the bulbar-onset ALS patients often showed a poorer prognosis with shorter survival time when compared with the spinal-onset patients. In the present study, we also observed a relatively short survival for bulbar-onset cases, although not statistically significant. A younger AAO in Chinese patients with ALS, as well as a small sample size of bulbar-onset patients in this study may be part of the reasons. Additionally, there may be other disease-modifying factors which remain unclear now. Secondly, although the electrophysiological tests were performed by the same board-certified neurologist, we have not assessed the intra- and inter-rater variability of median nerve NI. Additionally, some other prognostic factors for survival, such as pulmonary function parameters (FVC) were not included in our study. Finally, as a retrospective case series study, we could not compare the median nerve and ulnar nerve NI. We assume that it will be helpful to design a prospective study to compare their prognostic value in future.

In summary, we found that median nerve NI, together with older AAO, shorter diagnostic delay, higher ΔALSFRS-R were correlated with short survival of ALS. The median nerve NI is another useful predictor and measure of the outcome of ALS.

## Data Availability Statement

The original contributions presented in the study are included in the article/Supplementary Material, further inquiries can be directed to the corresponding author/s.

## Ethics Statement

The studies involving human participants were reviewed and approved by Ethics Board of the First Affiliated Hospital of Fujian Medical University. The patients/participants provided their written informed consent to participate in this study.

## Author Contributions

W-JC, NW, and Q-JZ: study concept and design. L-QX, WH, Q-FG, L-LL, G-RX, and Q-JZ: acquisition and interpretation of data. Q-JZ, L-QX, and WH: drafting of the manuscript. Q-JZ, W-JC, and NW: critical revision of the manuscript for important intellectual content. Q-JZ and NW: obtaining of funding and study supervision. All authors read and approved the final manuscript.

## Conflict of Interest

The authors declare that the research was conducted in the absence of any commercial or financial relationships that could be construed as a potential conflict of interest.
