# Supplementary material for: Median Nerve-Neurophysiological Index Correlates With the Survival of Patients With Amyotrophic Lateral Sclerosis
Source: Front Neurol. 2020 Oct 22;11:570227. doi: 10.3389/fneur.2020.570227 (PMC7642643; doi:10.3389/fneur.2020.570227)
Supplement: Supplementary file 1 [file Table_1.DOCX]

Supplementary Material


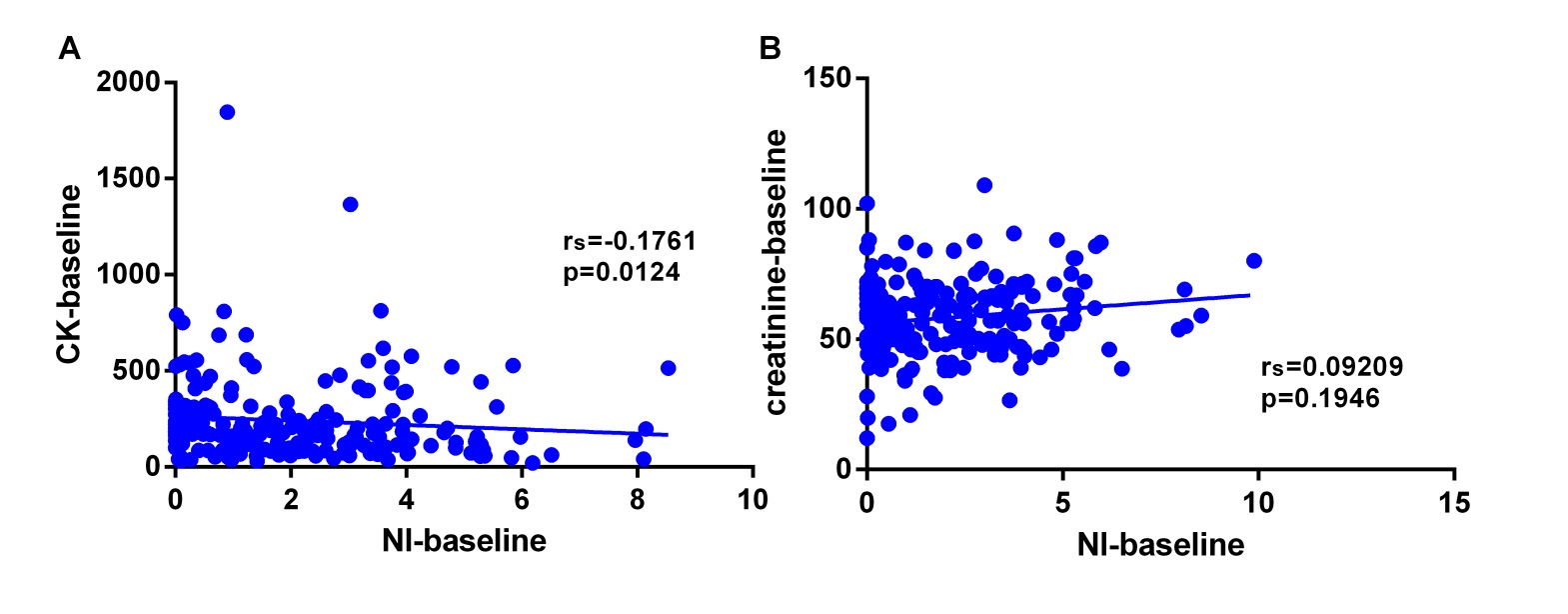


**Supplementary Figure 1.** (A) Correlation between median nerve-NI at baseline and serum CK levels at baseline (rs=-0.1761, *P*=0.0124). (B) Correlation between median nerve-NI at baseline and serum creatinine levels at baseline (rs=0.09209, *P*=0.1946).

**Supplementary Table 1.** The clinical features of ALS patients based on the median nerve-NI.

| Variable |  | median nerve-NI | | χ^2^ | *P* |
| --- | --- | --- | --- | --- | --- |
|  |  | ≤2.15 | >2.15 |  |  |
| gender |  |  |  | 0.156 | 0.693 |
|  | male | 83 | 64 |  |  |
|  | female | 49 | 42 |  |  |
| site of onset |  |  |  | 18.628 | <0.001 |
|  | bulbar-onset | 12 | 33 |  |  |
|  | spinal-onset | 120 | 73 |  |  |
| diagnostic category |  |  |  | 0.022 | 0.989 |
|  | definite | 87 | 69 |  |  |
|  | probable | 35 | 29 |  |  |
|  | possible | 10 | 8 |  |  |
| use of riluzole |  |  |  | 0.25 | 0.617 |
|  | no | 45 | 32 |  |  |
|  | yes | 87 | 74 |  |  |
| BMI |  |  |  | 7.273 | 0.026 |
|  | 18.5~24 | 96 | 64 |  |  |
|  | <18.5 | 21 | 16 |  |  |
|  | >24 | 15 | 26 |  |  |
| diagnostic delay |  |  |  | 1.178 | 0.278 |
|  | >12 m | 50 | 33 |  |  |
|  | ≤12 m | 82 | 73 |  |  |
| age of onset |  |  |  | 1.557 | 0.212 |
|  | ≤55 y | 59 | 56 |  |  |
|  | >55 y | 73 | 50 |  |  |
| ALSFRS-R |  |  |  | 10.249 | 0.001 |
|  | >38 | 79 | 84 |  |  |
|  | ≤38 | 53 | 22 |  |  |
| ΔALSFRS-R |  |  |  | 7.178 | 0.007 |
|  | ≤0.8 | 63 | 69 |  |  |
|  | >0.8 | 69 | 37 |  |  |
